# Association Between Cardiovascular Health Status and Healthcare Utilization in a Large Integrated Healthcare System

**DOI:** 10.1016/j.focus.2024.100213

**Published:** 2024-02-24

**Authors:** Irvin Lien, Howard Moffet, Jennifer Liu, Andrew Karter, Matthew Solomon, Alan Go, Khurram Nasir, Stephen Sidney, Jamal Rana

**Affiliations:** 1 Department of Internal Medicine, Kaiser Permanente Oakland Medical Center, Oakland, California; 2 Department of Cardiology, Kaiser Permanente Oakland Medical Center, Oakland, California; 3 Division of Research, Kaiser Permanente Northern California, Oakland, California; 4 Division of Cardiovascular Medicine, Houston Methodist Hospital, Houston, Texas

**Keywords:** Cardiovascular Health, expenditures, preventative medicine, healthcare utilization, healthcare cost, cardiovascular disease

## Abstract

**Introduction:**

The American Heart Association Life's Simple 7 schema can be used to categorize patients’ cardiovascular health status as poor, intermediate, or ideal on the basis of smoking, BMI, physical activity, dietary patterns, blood pressure, cholesterol, and fasting blood sugar. This study examined the association between cardiovascular health status and subsequent healthcare utilization.

**Methods:**

This was an observational cohort study of adults from an integrated healthcare delivery system—Kaiser Permanente Northern California—that had outpatient care between 2013 and 2014. Patients were categorized by American Heart Association cardiovascular health status: poor, intermediate, or ideal. Individual-level healthcare utilization and costs in 2015 were accumulated for each patient and compared across the 3 cardiovascular health categories and stratified by age groups.

**Results:**

A total of 991,698 patients were included in the study. A total of 194,003 (19.6%) were aged 18–39 years; 554,129 (55.9%) were aged 40–64 years; and 243,566 (24.6%) were aged ≥65 years. A total of 259,931 (26.2%) had ideal cardiovascular health; 521,580 (52.6%) had intermediate cardiovascular health; and 210,187 (21.2%) had poor cardiovascular health. Healthcare utilization measured by average relative cost per patient increased monotonically across age categories (*p*<0.001). In addition, cardiovascular health category was inversely associated with lower cost in each age group (*p*<0.001).

**Conclusions:**

Adults who were younger and had more ideal cardiovascular health had relatively lower healthcare costs across age groups. Interventions to promote better cardiovascular health may improve patient outcomes and reduce overall healthcare expenditures.

## HIGHLIGHTS

• The American Heart Association has identified 7 cardiovascular risk factors as targets to reduce the risk of cardiovascular disease.
• These 7 factors are: smoking, BMI, physical activity, dietary patterns, blood pressure, cholesterol, and fasting blood sugar.
• Patients with ideal cardiovascular health (CVH) are associated with decreased healthcare utilization.
• Older adults with poorer CVH had significantly higher relative healthcare costs.
• Further studies are needed to investigate whether interventions that improve CVH can reduce overall healthcare expenditures.

## INTRODUCTION

Cardiovascular disease (CVD) is the leading cause of mortality and morbidity worldwide ^1^ Every decade, the American Heart Association (AHA) publishes an Impact Goal, with intervention targets aimed at reducing the burden of CVD. The Impact Goal established at the AHA meeting in 2010 was to reduce deaths from CVDs and stroke by 20% by 2020. This would be accomplished by targeting 7 cardiovascular risk factors (Life's Simple 7): 4 modifiable behaviors (smoking, BMI, physical activity, dietary patterns) and 3 biometric measures (blood pressure, cholesterol, and fasting blood sugar).^2^ Each cardiovascular health (CVH) metric was defined by thresholds of ideal, intermediate, and poor. Ideal CVH has been associated with lower all-cause and cardiovascular mortality.^3^, ^4^, ^5^, ^6^

Although the primary goal of the Impact Goal was to reduce the burden of CVDs, it is also of interest to assess CVH metrics and healthcare utilization. Smaller studies have noted the absence of atherosclerotic cardiovascular disease (ASCVD) and that favorable cardiovascular risk factors are associated with lower medical expenditures.^7^ In this study, the authors examined the association between CVH metrics and healthcare utilization in a large integrated healthcare delivery system. They hypothesized that patients who are younger and have ideal CVH will have less healthcare system utilization and lower healthcare spending than those with intermediate or poor CVH.

## METHODS

### Study Population

This was an observational cohort study of adults (aged ≥18 years) who were Kaiser Permanente Northern California (KPNC) members; had any outpatient appointment within a Kaiser Permanente office during any point between January 1, 2013 and January 1, 2015; had documented race; and had documented CVH metrics. IRB approval was obtained internally by the Kaiser Permanente Division of Research (IRB Reference Number 1278953). IRB approval letters are FWA#00002344 and IRB#00001045. This data collection method was the same as in a prior study that assessed the association of CVH metrics and incidence of stroke, fatal coronary artery disease, or ASCVD.^8^

### Measures

Inclusion criteria were patients with no documented history of ASCVD and electronic health record data for all 6 of the 7 AHA CVH metrics utilized in this study (smoking status, physical activity, BMI, blood pressure, total cholesterol, and fasting blood glucose; diet score was not routinely collected for patients and thus not included in the study). The smoking status and amount of physical activity were metrics self-reported by patients. Each metric was categorized as poor (0), intermediate (1), and ideal (2) according to the modified AHA definitions as outlined in the 2020 Impact Goal. The summed composite score resulted in an overall CVH assessment score: poor CVH (0–6), intermediate CVH (7–9), or ideal CVH (10–12). In addition, patients were categorized by age: 18–39, 40–64, or ≥65 years.

### Statistical Analysis

Individual-level healthcare utilization and costs during the entirety of 2015 were extracted from administrative databases and included inpatient hospital, outpatient clinic, pharmacy, emergency department, and other miscellaneous KPNC service healthcare costs. The authors calculated the average cost per patient for the total population and then calculated the average cost per patient by overall CVH assessment score and stratified by the age groups. A chi-square test was utilized to assess significant cost differences between the different groups.

## RESULTS

**Figure 1:**
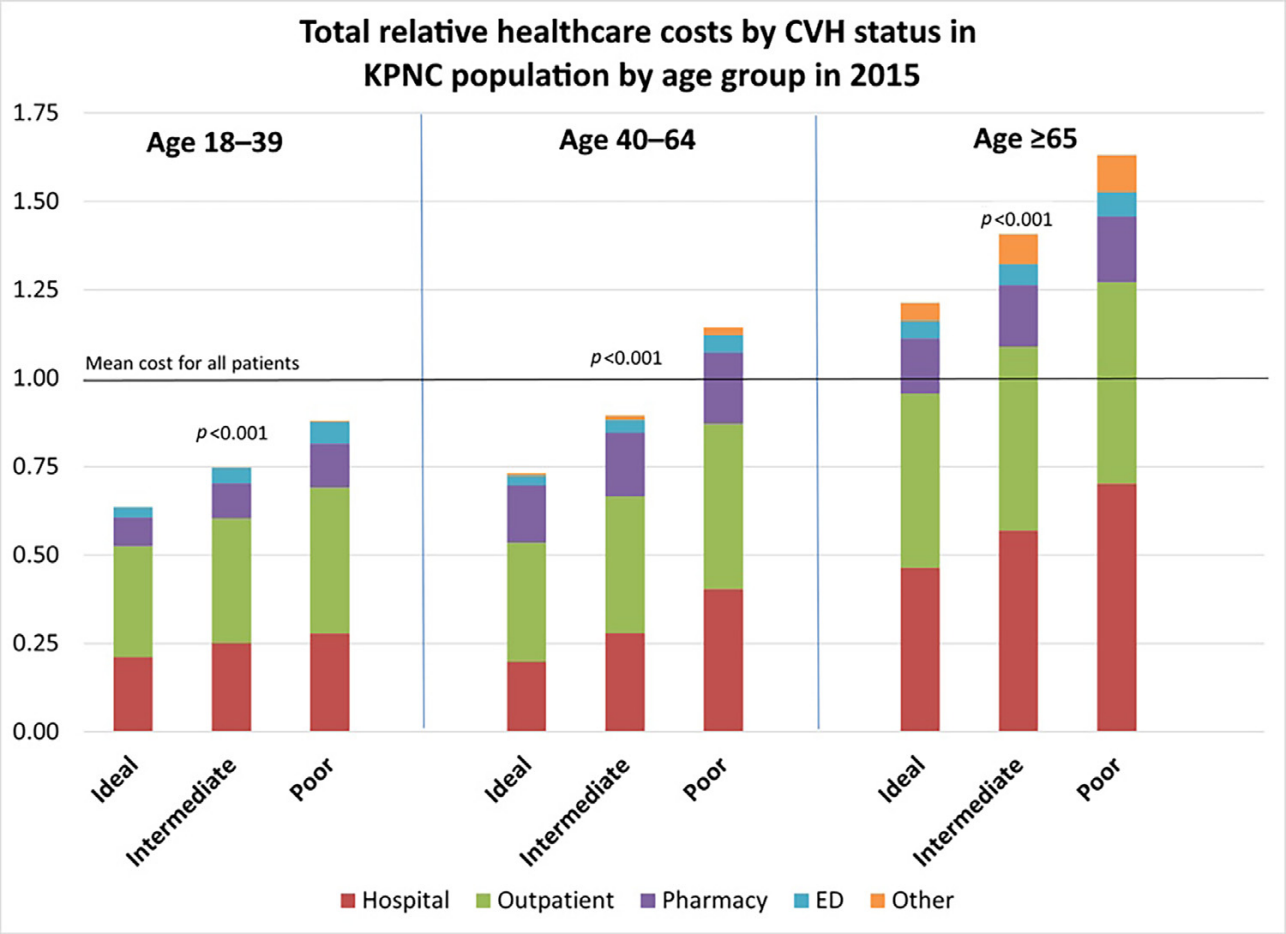
Relative healthcare costs per patient by cardiovascular health categories in KPNC population by age groups in 2015. Ages are in years. ED, emergency department; KPNC, Kaiser Permanente Northern California.

**Figure 2:**
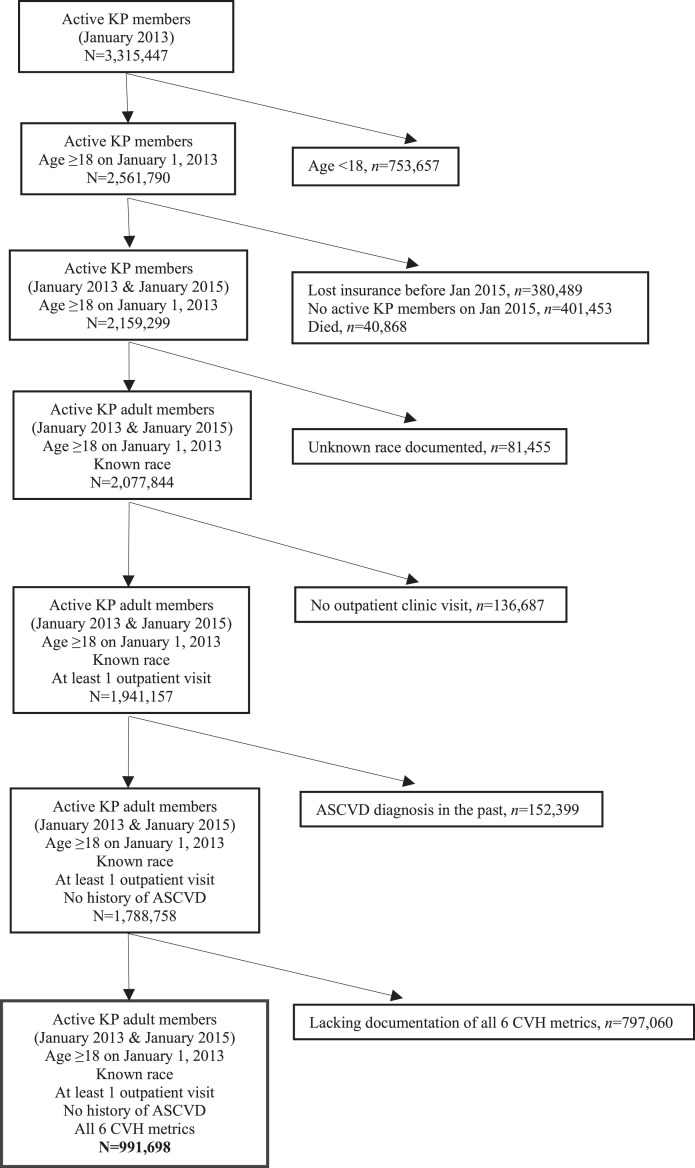
KP members who met inclusion criteria for data analysis. KP, Kaiser Permanente.

Of the over 3.3 million adult KPNC members assessed for inclusion for data analysis, a total of 991,698 KPNC members were included on the basis of this study's inclusion and available metrics (Figure 2). The population was diverse and comprised 564,851 (57.0%) women as well as 211,144 (21.3%) Asian/Pacific Islander; 159,873 (16.1%) Hispanic; and 67,049 (6.8%) Black patients. A total of 194,003 (19.6%) were aged 18–39 years, with a mean age of 31.8 years; 554,129 (55.9%) were aged 40–64 years, with a mean age of 53.0 years; and 243,566 (24.6%) were aged ≥65 years, with a mean age of 72.9 years. On the basis of the patient's overall CVH metrics, 259,931 (26.2%) had ideal CVH; 521,580 (52.6%) had intermediate CVH; and 210,187 (21.2%) had poor CVH (Table 1). On average, patients aged 18–39 years had relatively lower healthcare costs than the total average. Patients aged 40–64 years with ideal CVH and intermediate CVH also had relatively lower healthcare costs than the total average. Patients aged 40–64 with years had poor CVH, and all patients aged ≥65 years had relatively higher average healthcare costs. The overall cost increased monotonically across age categories (*p*<0.001). In addition, ideal CVH was associated with lower cost in each age group than both intermediate CVH and poor CVH (*p*<0.001). Each age group had a significantly different average cost, with younger patients having lower costs overall (Figure 1).

**Table 1:** Demographics and Cardiovascular Health Metrics of 991,698 Kaiser Permanente Northern California Members in 2015 (Overall and by Age Group).

Demographics		Overall	Aged 18–39 years	Aged 40–64 years	Aged ≥65 years
		*n* (%)	*n* (%)	*n* (%)	*n* (%)
Sex	Women	564,851 (57.0)	112,129 (57.8)	309,917 (55.9)	142,805 (58.6)
	Men	426,847 (43.0)	81,874 (42.2)	244,212 (44.1)	100,761 (41.4)
Age, year	Mean (SD)	53.8 (15.1)	31.8 (5.9)	53.0 (7.0)	72.9 (6.3)
	18–39	194,003 (19.6)	194,003 (100)	0	0
	40–64	554,129 (55.9)	0	554,129 (100)	0
	≥65	243,566 (24.6)	0	0	243,566 (100)
Race	White	514,902 (51.9)	70,835 (36.5)	284,312 (51.3)	159,755 (65.6)
	Black	67,049 (6.8)	12,935 (6.7)	40,978 (7.4)	13,136 (5.4)
	Hispanic	159,873 (16.1)	46,788 24.1)	89,974 (16.2)	23,111 (9.5)
	Asian/PI	211,144 (21.3)	55,194 (28.4)	119,522 (21.6)	36,428 (15.0)
	Other/mixed	38,730 (3.9)	8,251 (4.3)	19,343 (3.5)	11,136 (4.6)
Metrics					
Smoking					
0, current smoker	(0) poor	68,016 (6.9)	14,694 (7.6)	42,556 (7.7)	10,766 (4.4)
1, quit ≤12 months ago	(1) intermediate	11,261 (1.1)	3,578 (1.8)	6,303 (1.1)	1,380 (0.6)
2, never smoker or quit >12 months ago	(2) ideal	912,421 (92.0)	175,731 (90.6)	505,270 (91.2)	231,420 (95.0)
BMI					
0, ≥30 kg/m^2^	(0) poor	345,099 (34.8)	69,847 (36.0)	207,135 (37.4)	68,117 (28.0)
1, 25.0–29.9 kg/m^2^	(1) intermediate	347,714 (35.1)	60,156 (31.0)	195,762 (35.3)	91,796 (37.7)
2, <25 kg/m^2^	(2) ideal	298,885 (30.1)	64,000 (33.0)	151,232 (27.3)	83,653 (34.3)
Physical activity: exercise/week					
0, 0 minutes	(0) poor	341,330 (34.4)	63,358 (32.7)	186,560 (33.7)	91,412 (37.5)
1, 1–149 minutes	(1) intermediate	263,668 (26.6)	54,580 (28.1)	149,462 (27.0)	59,626 (24.5)
2, ≥150 minutes	(2) ideal	386,700 (39.0)	76,065 (39.2)	218,107 (39.4)	92,528 (38.0)
Cholesterol					
0, ≥240 mg/dL	(0) poor	91,121 (9.2)	10,685 (5.5)	60,373 (10.9)	20,063 (8.2)
1, 200-239 mg/dL	(1) intermediate	272,893 (27.5)	40,715 (21.0)	170,393 (30.8)	61,785 (25.4)
2, <200 mg/dL	(2) ideal	627,684 (63.3)	142,603 (73.5)	323,363 (58.4)	161,718 (66.4)
BP					
0, SBP ≥140 or DBP ≥90 mmHg	(0) poor	128,872 (13.0)	13,763 (7.1)	70,785 (12.8)	44,324 (18.2)
1, SBP 129–130 mmHg or DBP 80–89 mmHg	(1) intermediate	534,493 (53.9)	86,203 (44.4)	302,812 (54.6)	145,478 (59.7)
2, SBP <120 mmHg and DBP <80 mmHg	(2) ideal	327,333 (33.1)	94,037 (48.5)	180,532 (32.6)	53,764 (22.1)
Glucose					
0, >125 mg/dL fasting	(0) poor	94,177 (9.5)	7,106 (3.7)	55,525 (10.0)	31,546 (12.9)
1, 100–125 mg/dL fasting	(1) intermediate	278,939 (28.1)	25,316 (13.0)	162,648 (29.4)	90,975 (37.4)
2, <100 mg/dL fasting	(2) ideal	618,582 (62.4)	161,581 (83.3)	335,956 (60.6)	121,045 (49.7)
Cumulative cardiovascular health					
	Poor (0–6)	210,187 (21.2)	25,619 (13.2)	131,301 (23.7)	53,267 (21.9)
	Intermediate (7–9)	521,580 (52.6)	92,912 (47.9)	290,051 (52.3)	138,617 (56.9)
	Ideal (10–12)	194,003 (19.6)	75,472 (38.9)	132,777 (24.0)	51,682 (21.2)

BP, blood pressure; DBP, diastolic blood pressure; SBP, systolic blood pressure.

## DISCUSSION

In this study using data from a large integrated healthcare system with a diverse population, better CVH as defined by the 2020 Impact Goal was associated with lower healthcare utilization. This association was consistent among patients within each age group: ages 18–39, ages 40–64, and age ≥65 years.

These data had findings similar to those of another smaller population study called the “REasons for Geographic And Racial Differences in Stroke” study.^9^ The “REasons for Geographic And Racial Differences in Stroke” study included 6,262 Medicare patients aged >65 years, residing in the southeastern U.S. The study concluded that patients with a higher number of CVD metrics within the ideal range based on the AHA 2020 Impact Goal were associated with lower inpatient and outpatient healthcare utilization. Another analysis of 15,651 patients derived from the 2012 Medical Expenditure Panel Survey found that patients with more favorable CVH had significantly lower medical expenditure and healthcare utilization with and without CVD.^10^ An older longitudinal study called the Chicago Heart Association Detection Project Industry (1967–1973), which included 17,195 participants similarly, saw that poor CVH led to increased healthcare costs over time with more frequent acute inpatient visits.^11^ There are a few other studies that have also investigated how CVDs can affect healthcare utilization, for example, high utilization of health care for adults with congenital heart disease^12^ and higher healthcare costs in treatment of CVDs in patients with Type 2 diabetes.^13^

The present study is one of the few studies to include analysis from a large integrated healthcare system. It is uniquely large and includes nearly 1 million patients. In addition, the authors analyzed a racially and ethnically diverse population, including Asian/Pacific Islander, Hispanic, and Black individuals. The short-term follow-up of a year also avoids the need for long-term follow-up where patients can be lost or have time-varying changes to CVH. However, more research needs to be done to see whether improving CVH through preventative interventions can reduce the cost of health care and improve patient outcomes in a large integrated healthcare system long term.

### Limitations

There are a few limitations to this study. The authors did not have detailed data on dietary composition, so this metric was not included in the study. In addition, fine details on the intensity of physical activity and overall management of diabetes, hypertension, and hyperlipidemia were not measured, which would also impact overall CVH. Smoking status and physical activity are self-reported metrics and were up to patients to report truthfully. In 2022, the AHA made an addendum to include sleep characteristics as an eighth metric to measure, which was not collected.^14^ These data were only collected among patients within the KPNC healthcare system and did not include patients who did not have all 6 CVH metrics the study was measuring, thus excluding patients who did not routinely see their healthcare providers. The study took place in a large diverse population in Northern California; thus, results from this study may not apply outside of the region. Patients who did not meet theinclusion criteria are more likely to be patients who do not get routine follow-up and are at higher risk for having poorer healthcare literacy and poorer CVH metrics; thus, this study may include more patients with improved CVH metrics than what would have been expected. Finally, this is a broad evaluation of the correlation between CVH and healthcare utilization. Confounding variables outside of the outlined AHA health metrics, including SES, healthcare literacy, and other comorbidities, may also impact CVH but were not included in the study.

## CONCLUSIONS

This study, which includes a large real-world diverse population, found that older adults with poorer CVH had significantly higher relative healthcare costs. This persisted even when stratifying by different age groups. Future research can consider pragmatic trials to test the clinical and cost-effectiveness of prevention interventions to improve CVH, for example, longitudinal studies to assess whether improvements in CVH correlate with reduction in healthcare utilization. The hope is that prioritizing preventative interventions to improve CVH can help reduce overall healthcare spendings while also improving patient outcomes.

## CRediT authorship contribution statement

**Irvin Lien:** Writing – original draft. **Howard Moffet:** Conceptualization, Methodology, Formal analysis, Writing – review & editing. **Jennifer Liu:** Conceptualization, Methodology, Formal analysis. **Andrew Karter:** Data curation, Conceptualization, Formal analysis. **Matthew Solomon:** Conceptualization, Methodology, Formal analysis. **Alan Go:** Conceptualization, Methodology, Formal analysis. **Khurram Nasir:** Conceptualization, Methodology, Formal analysis. **Stephen Sidney:** Data curation, Formal analysis. **Jamal Rana:** Conceptualization, Methodology, Formal analysis, Supervision, Writing – review & editing.
